# The Application Value of Flow Cytometry‐Based Peripheral Blood Leukocyte Classification in Screening for Acute Leukemia and Posttreatment Monitoring

**DOI:** 10.1002/jcla.70206

**Published:** 2026-03-18

**Authors:** Penghua Yan, Lianfeng Wu, Xiangyang Lin, Hong Lu, Jingjing Qian

**Affiliations:** ^1^ Department of Clinical Laboratory Key Laboratory of Clinical Laboratory Diagnosis and Translational Research of Zhejiang Province The First Affiliated Hospital of Wenzhou Medical University Wenzhou Zhejiang China; ^2^ Zhejiang Key Laboratory of Intelligent Cancer Biomarker Discovery and Translation, First Affiliated Hospital Wenzhou Medical University Wenzhou Zhejiang China

**Keywords:** acute leukemia, flow cytometry, peripheral blood leukocyte classification, screening and monitoring

## Abstract

**Objective:**

Assess the application value of flow cytometry (FCM) for peripheral blood leukocyte (PBL) classification in acute leukemia (AL) screening and monitoring.

**Methods:**

Analyzed EDTA‐K2‐anticoagulated blood from 100 nonhematological patients, 42 newly diagnosed AL patients, and 50 posttreatment leukemia patients using FCM, blood cell analyzers (BCA), and manual microscopy.

**Results:**

FCM and BCA showed significant positive correlations in classifying and counting neutrophils, eosinophils, basophils, lymphocytes, and monocytes (*r* = 0.994, 0.671, 0.946, 0.987, and 0.849, respectively; *p* < 0.01). A significant positive correlation was observed between FCM and manual methods in counting blast cells (*r* = 0.882, *p* < 0.01). Using microscopy as the reference standard and a cutoff value of ≥ 1% blast cells, FCM demonstrated 100% sensitivity, 85.7% specificity, and 97.8% accuracy in detecting blast cells. The type of peripheral blood blast cells provided by FCM showed good consistency with the clinical diagnosis of leukemia (Kappa value = 0.834). Additionally, a positive correlation was found between the detection rates of bone marrow and peripheral blood blast cells using FCM in posttreatment patients with leukemia (*r*s = 0.860, *p* < 0.01).

**Conclusion:**

FCM‐based PBL classification is precise and efficient for detecting blasts, providing rapid subtype identification. Its strong correlation with marrow findings underscores its clinical utility in AL screening and monitoring.

## Graphical abstract

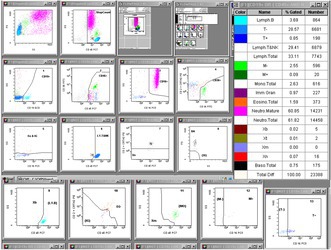

## 1 Introduction

In the clinical diagnosis and treatment of acute leukemia (AL), early screening primarily relies on peripheral blood leukocyte (PBL) counts [1]. Diagnosis is then confirmed through bone marrow morphology [2, 3]. To achieve the best therapeutic outcome, patients with confirmed AL need to review the bone marrow morphology assessments after induction chemotherapy. These evaluations are crucial for adjusting chemotherapy intensity based on the level of residual leukemic cells in the bone marrow and the degree of bone marrow hyperplasia [4, 5, 6]. However, bone marrow puncture often fails in acute myeloid leukemia (AML) patients due to short intervals between procedures and chemotherapy‐induced marrow suppression. Some studies have taken peripheral blood as the detection object. These studies suggest that small residual leukemic cells in peripheral blood are associated with the long‐term prognosis of patients with AML, suggesting that the existence of circulating blast immature cells in peripheral blood correlates with the persistence of tumor cells in bone marrow [7]. Therefore, it is imperative to pay close attention to the results of PBL classification in AL screening and posttreatment monitoring.

Currently, the two most common methods for PBL classification and counting are blood cell analyzers and morphological microscopy [8, 9]. While blood cell analyzers allow for automation and standardization, they are unable to accurately identify pathological cells. Abnormal graphics and alarm information still need confirmation by microscopy and other methods. Morphological microscopy is considered the “gold standard,” but it is labor‐intensive, time‐consuming, and requires trained professionals. Additionally, it is greatly affected by subjective factors, and the limited number of counting cells examined increases the risk of missing or misclassifying blast cells [10].

With the development of flow cytometry (FCM) technology, FCM in conjunction with commercialized combined antibody and automated analysis software has emerged. Therefore, the use of polychromatic, rapid FCM for PBL counting has become a growing trend [11, 12, 13]. This approach compensates for the limitations of blood cell analyzer and morphological microscopy, allowing for the classification of more leukocyte subsets [14, 15]. In this study, peripheral blood samples were analyzed using FCM, blood cell analyzer, and morphological microscopy to compare leukocyte classification results. The study verified the reliability of FCM in classifying and counting PBLs, particularly blast cells, and further compared these results with bone marrow blast cells to analyze their correlation and consistency, and discussed their application value in the screening of AL and posttreatment monitoring.

## 2 Materials and Methods

### 2.1 Research Object

This study included a total of 192 outpatient and inpatient patients from the First Affiliated Hospital of Wenzhou Medical University between January 2020 and July 2021. Of these, 100 patients had nonhematological diseases (61 males and 39 females, aged 13–86 years, with a median age of 58 years), and 92 patients were diagnosed with AL (37 males and 55 females, aged 15–81 years, with a median age of 55 years). Based on the 2022 WHO classification criteria for hematopoietic and lymphoid tissue tumors [16], the distribution of 92 cases of AL is shown in Table 1. This study was approved by the Ethics Committee Clinical Research of the First Affiliated Hospital of Wenzhou Medical University (China).

**TABLE 1:** Distribution of AL types.

Disease type	Number
B‐lymphoblastic leukemia (B‐ALL)	14
Chronic myeloid leukemia to B‐ALL	1
T‐lymphoblastic leukemia (T‐ALL)	3
Acute promyelocytic leukemia (APL)	3
Acute myeloid leukemia (AML)	64
Myelodysplastic syndrome to AML	2
Chronic myeloid leukemia to AML	1
Chronic myeloid leukemia to B‐ALL	1
Chronic myelomonocytic leukemia to AML	1
Lymphoma leukemia (LCL)^a^	2

^a^ LCL should not actually be classified as AL, but due to its unique expression in FCM, it should be analyzed together.

### 2.2 Instruments and Reagents

The instruments used in this study included XE2100 fully automatic blood cell analyzer (Sysmex, Japan) with its supporting reagents and quality control materials, the SP‐1000i automatic slide staining machine (Sysmex, Japan), a binocular microscope (Olympus, Japan), and FC500 flow cytometer (Beckman Coulter, USA) with its supporting sheath and cleaning solution. Flow combination reagents included CD36‐FITC, CD2‐PE, CD294‐PE, CD19‐ECD, CD16‐PC5, and CD45‐PC7 antibodies. Additional reagents included calibration microspheres (Flow Set), hemolytic agents, and quality control microspheres (Flow Check).

### 2.3 Blood Cell Analyzer Detection

Daily quality control was conducted in the laboratory using matching quality control products. During the experimental process, the inter‐laboratory quality control results were qualified. According to the requirements of the laboratory, various indicators were measured, including white blood cell counts, and sampling was completed within 2 h.

### 2.4 FCM Detection

**FIGURE 1:**
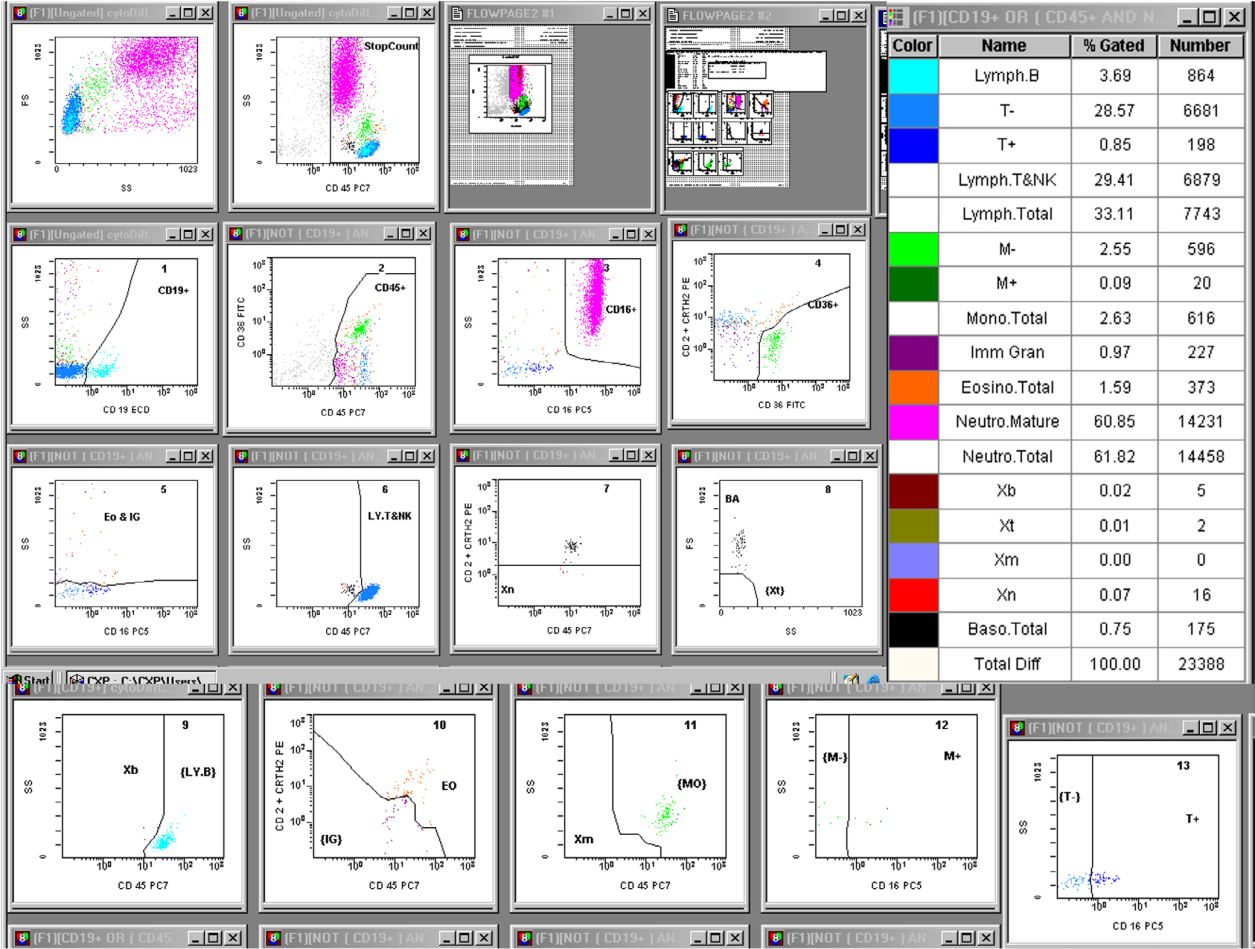
Example of leukocyte classification results by FCM. The 16 cell populations are displayed in different colors according to the set gate logic.

According to the manufacturer's instructions, 10 μL of FCM combination reagents (anti‐CD36‐FITC, anti‐CD2‐PE, anti‐CD294‐PE, anti‐CD19‐ECD, anti‐CD16‐PC5, and anti‐CD45‐PC7) were mixed with 100 μL of whole blood. The mixture was immediately vortexed for 3–5 s and incubated at room temperature in the dark for 15–20 min. Then, 1 mL of hemolysin was added, vortexed for 3–5 s, and incubated at room temperature in the dark for 15 min before being analyzed. Reagents and fluorescent microspheres provided in the FCM were used to calibrate and compensate the instrument before the experiment. The sample was placed on the FCM rack, and the CXP automatic analysis software was used to measure the sample. No less than 20,000 cells were collected in each test tube. The gating strategy was adjusted. Based on the different expression types and fluorescence intensities of various white blood cell surface antigens in peripheral blood, along with the light scattering characteristics of different cells, a multiple logic gating method was applied to divide the cells into 16 white blood cell subgroups: total lymphocytes B lymphocytes, total T/natural killer lymphocytes, cytotoxic T/natural killer lymphocytes, noncytotoxic T lymphocytes, total monocytes, pro‐inflammatory monocytes, noninflammatory monocytes, mature neutrophils, eosinophils, basophils, immature granulocytes, total blast cells, blast T cells, blast B cells, and blast non‐T non‐B cells (Figure 1). Specimen testing data were collected, and testing was completed within 4 h after collection.

### 2.5 Morphological Microscopy (Manual Method)

After thoroughly mixing the specimen, a blood smear was prepared using a fully automatic slide staining instrument, followed by Wright‐Giemsa staining. A qualified examiner, who had undergone regular morphological training and assessment, performed a microscopic examination of 100 white blood cell classifications and recorded the results. If blast cells were not detected by manual methods but were identified by FCM classification technology, the findings were reviewed by an experienced bone marrow cell morphology inspector (with over 5 years of full‐time experience).

### 2.6 Morphological Examination of Bone Marrow Cells

Bone marrow smears were extracted from patients, stained with Wright‐Giemsa staining, and classified and counted for 200 cells under an oil microscope. For newly diagnosed patients, biochemical staining techniques, including peroxidase staining (POX), specific lipase staining (AS‐DCE), nonspecific esterase staining (NAE/NaF), and periodic acid Schiff staining (PAS), were performed to determine the type of AL.

### 2.7 Statistical Analysis

Statistical analysis was performed using SPSS 26.0 software. Bivariate Pearson correlation analysis was conducted to compare the two classification and counting methods. The rates were compared using a paired four‐cell table data chi‐square test. Kappa consistency analysis was used to compare leukemia types, and Spearman correlation analysis was performed on the detection rate of blast cells. A *p*‐value below 0.05 was considered statistically significant.

## 3 Result

### 3.1 Comparison of the Correlation Between FCM and Hemocytometer Methods for PBL Classification and Counting

**FIGURE 2:**
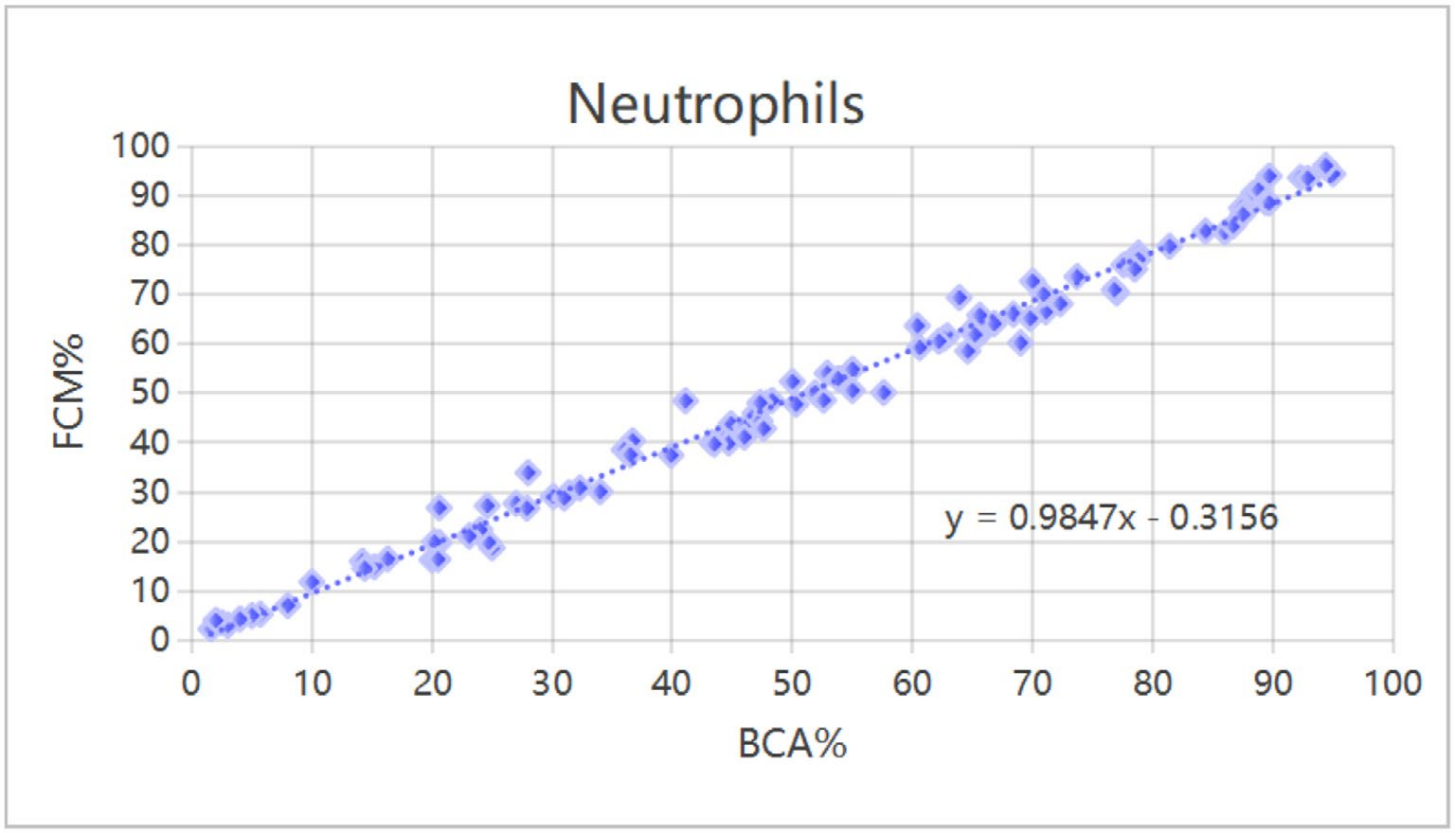
Correlation analysis of neutrophils counted by FCM and hematology analyzer method. A total of 100 samples were included in this study. The scatter plot reflects the distribution relationship between the percentage of neutrophils detected by hematology analyzer (*x*‐axis, scale: 0%–100%) and the percentage detected by flow cytometry (*y*‐axis, scale: 0%–100%). The dashed line represents the linear regression fitting trend, with the fitting equation *y* = 0.9847*x*–0.3156. Tested by Pearson product–moment correlation analysis, there was a significant correlation between the percentage of neutrophils detected by hematology analyzer and FCM (*r* = 0.994, *p* < 0.01).

**FIGURE 3:**
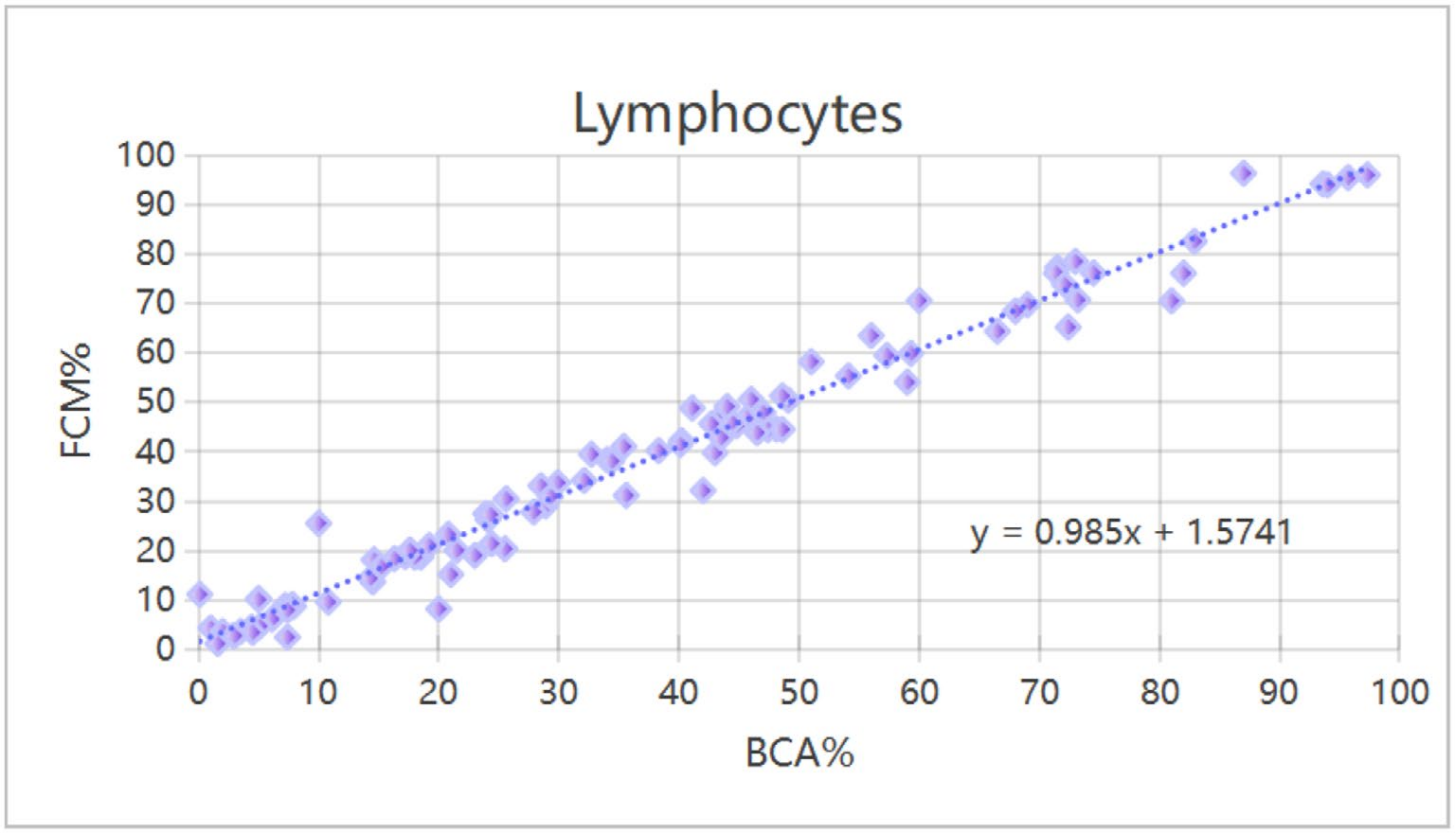
Correlation analysis of lymphocytes counted by FCM and hematology analyzer method. A total of 100 samples were included in this study. The scatter plot reflects the distribution relationship between the percentage of lymphocytes detected by hematology analyzer (*x*‐axis, scale: 0%–100%) and the percentage detected by flow cytometry (*y*‐axis, scale: 0%–100%). The dashed line represents the linear regression fitting trend, with the fitting equation *y* = 0.985*x* + 1.5741. Tested by Pearson product–moment correlation analysis, there was a significant correlation between the percentage of lymphocytes detected by hematology analyzer and FCM (*r* = 0.987, *p* < 0.01).

Neutrophils, lymphocytes, monocytes, eosinophils, and basophils were counted. A significant correlation was found between FCM and hematology analyzer, with R values of 0.994, 0.987, 0.849, 0.671, and 0.946 respectively (*p* < 0.01; Table 2). The correlation for neutrophils and lymphocytes was particularly significant, as shown in Figures 2 and 3.

**TABLE 2:** Correlation analysis between FCM and hematology analyzer method.

Category	Regression equation	*r*
Neutrophils	*y* = 0.9847*x* −0.3156	0.994*
Lymphocyte	*y* = 0.9850*x* + 1.5741	0.987*
Monocyte	*y* = 0.7767*x* + 0.9977	0.849*
Eosinophils	*y* = 0.7423*x* + 1.2091	0.671*
Basophils	*y* = 1.0971*x* + 0.3237	0.946*

* *p* < 0.01.

### 3.2 Comparison of the Correlation Between FCM and Manual Counting of Peripheral Blood Blast Cells

**FIGURE 4:**
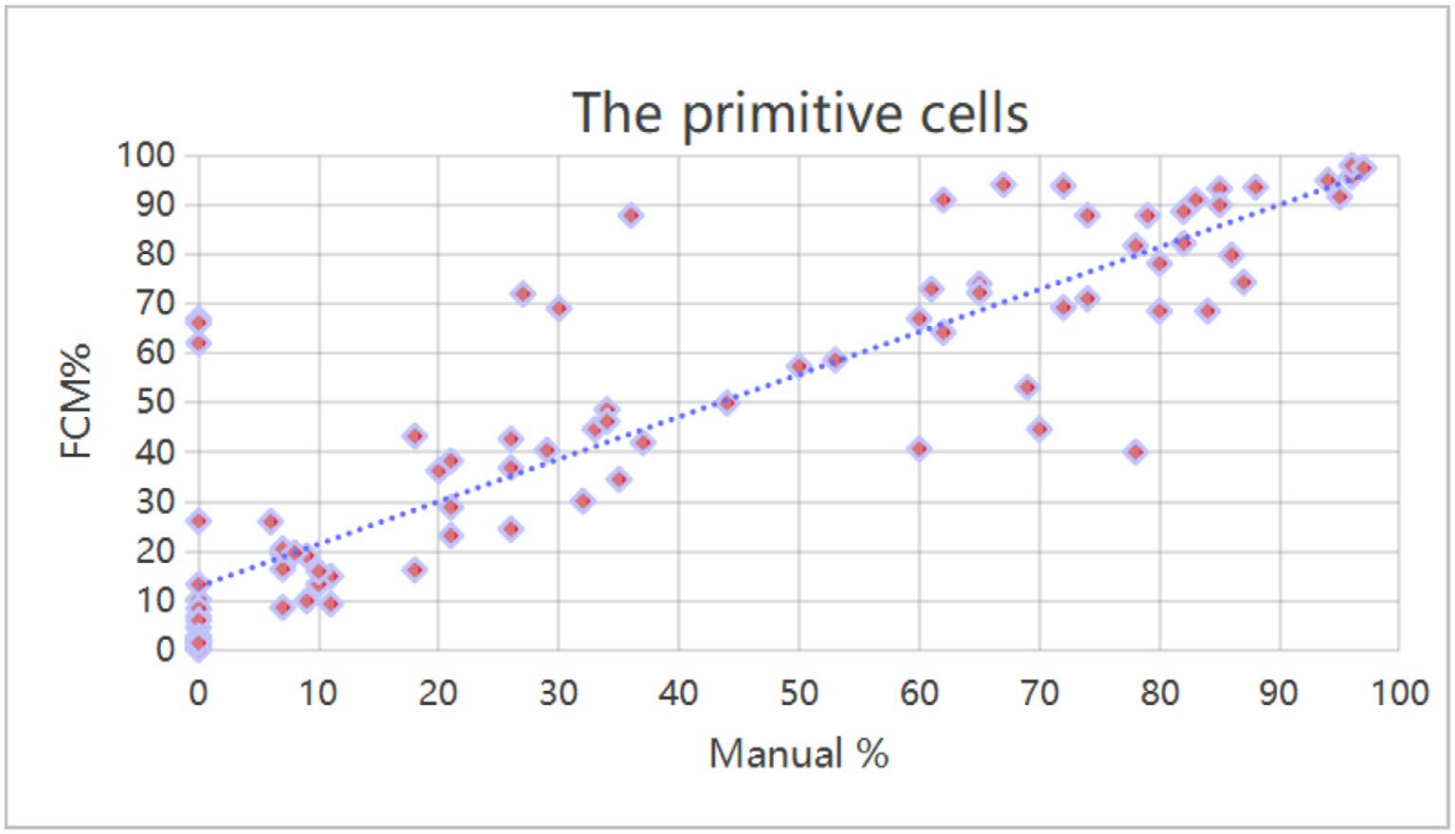
Correlation analysis of peripheral blood blast cells counted by FCM and manual methods. A total of 92 samples were included in this study. The scatter plot reflects the distribution relationship between the percentage of blast cells by manual counting (Manual%, *x*‐axis, scale: 0%–100%) and the percentage detected by flow cytometry (FCM%, *y*‐axis, scale: 0%–100%). The dashed line represents the linear regression fitting trend. Tested by Pearson product–moment correlation analysis, there was a significant correlation between the percentage of blast cells by manual counting and FCM% (*r* = 0.882, *p* < 0.01).

Peripheral blood blast cells were counted using both methods, and a significant correlation was observed between FCM and manual method (*r* = 0.882, *p* < 0.01), as shown in Figure 4.

### 3.3 Sensitivity and Specificity of FCM for Detecting Peripheral Blood Blast Cells

Using morphological microscopy (manual method) as the standard, blast cells were detected in 78 of the 92 samples (positive rate: 84.7%). When using FCM with a threshold of ≥ 1% for blast cells [17], 80 cases were detected (positive rate: 86.9%). The sensitivity was 100%; the specificity was 85.7%; and the accuracy was 97.8%, as shown in Table 3.

**TABLE 3:** Sensitivity and specificity analysis of FCM for detection of peripheral blood blast cells.

	Microscopic examination (+)	Microscopic examination (−)	Total
FCM (+)	68 + 10^a^	2	80
FCM (−)	0	12	12
Total	78	14	92

^a^ +10 indicates that there were 10 cases where blast cells were not detected by manual methods but were detected by FCM classification technology. After review by a senior morphologist, it was confirmed that blast cells were detected, and therefore both methods were used to detect them.

### 3.4 Consistency Analysis Between Blast Cell Types Provided by FCM and Clinically Diagnosed Leukemia Types

According to FCM, the blast cells were classified into blast B lymphocytes (XB), blast T lymphocytes (XT), blast monocytes (XM) and non‐T non‐B blast cells (xn). XB was associated with B‐ALL, XT with T‐ALL, and xm/xn/xm + xn with AML. The consistency of 42 initially diagnosed samples was analyzed. However, three cases of acute promyelocytic leukemia were excluded from the analysis, as they were classified as immature granulocytes rather than blast cells. Therefore, the kappa value for the remaining 38 samples was 0.834, as shown in Table 4.

**TABLE 4:** Analysis of concordance between primary cell types provided by FCM and clinically diagnosed leukemia types blast cells.

Number of cases		Clinical diagnostic type			Kappa value	*U*	*p*
38		AML	B‐ALL	T‐ALL	0.834	6.567	< 0.01
Peripheral blood blast cell types	Not detected ≥ 1%	1	0	0			
	Xn	30	0	0			
	Xm	0	0	0			
	Xt	1	0	1			
	Xb	0	5	0			

### 3.5 Comparison of the Correlation Between FCM Detection Rates of Peripheral Blood Blast Cells and Bone Marrow Blast Cells in Patients With Posttreatment Follow‐Up Visits

**FIGURE 5:**
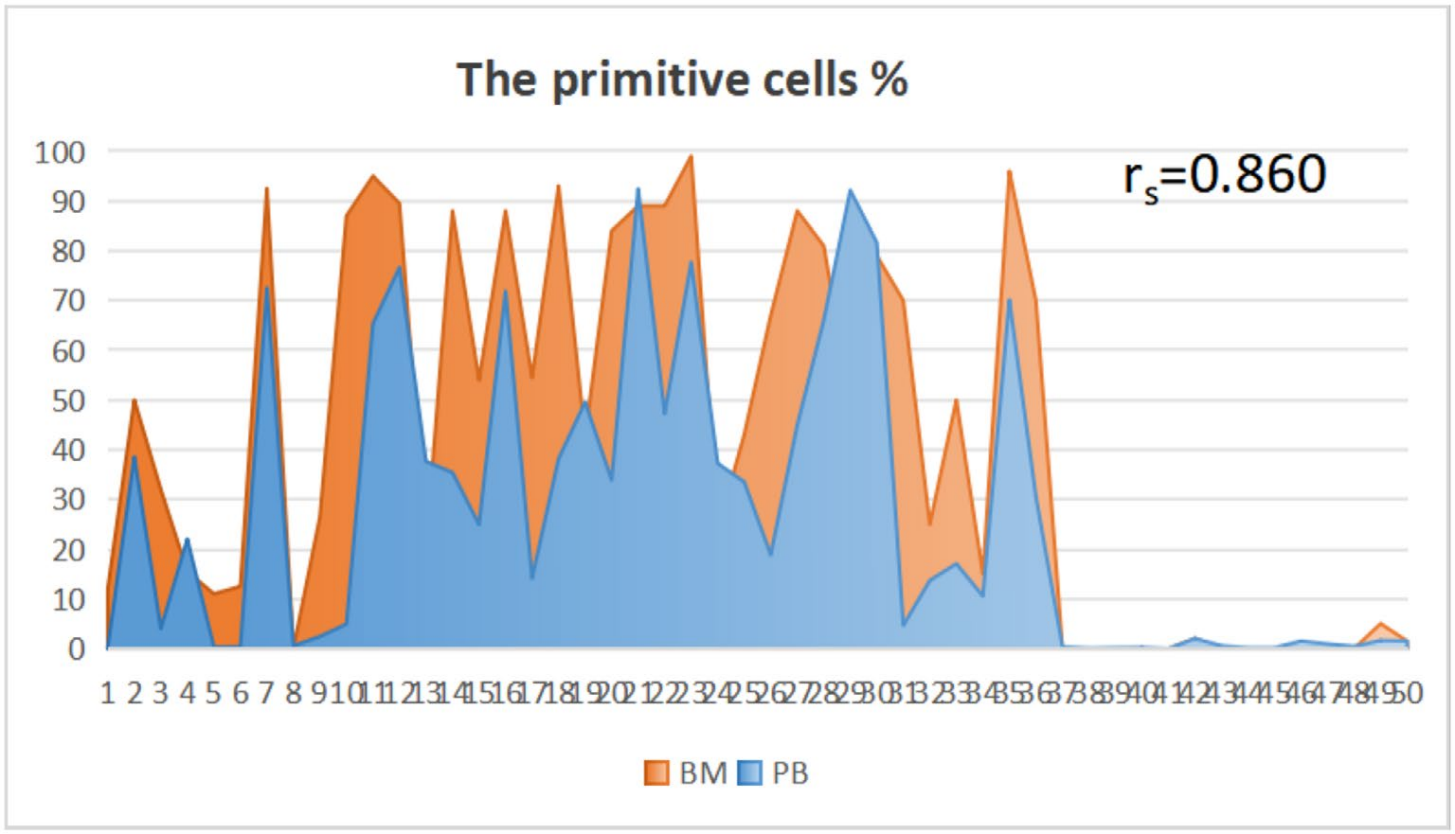
Distribution and Correlation analysis of the detection rate of peripheral blood blast cells and bone marrow blast cells by flow‐through assay in patients who returned to the clinic after treatment. A total of 50 samples were included in this study. The column chart reflects the distribution characteristics of blast cell percentages in bone marrow (BM, orange) and peripheral blood (PB, blue). The *y*‐axis represents the percentage of blast cells (scale: 0%–100%), and the *x*‐axis represents the sample numbers (1–50). Tested by Spearman's rank correlation analysis, there was a significant correlation between the blast cell percentages in bone marrow and peripheral blood (*r*s = 0.860, *p* < 0.01).

Morphological microscopy was used to count the bone marrow blast cells of 50 patients with leukemia posttreatment, and the correlation FCM detection rates of peripheral blood blast cells were analyzed. Spearman analysis showed a significant correlation between the two methods (*r*s = 0.860, *p* < 0.01), as shown in Figure 5.

## 4 Discussion

As a malignant tumor of the hematopoietic system, AL poses a severe threat to human life and health. Multiparameter FCM can recognize cells through specific monoclonal antibody markers. Currently, FCM is mainly used for immunophenotyping of leukemia in bone marrow [18] and is considered a standard for hematological analysis and leukemia classification. In recent years, FCM has gradually been applied to peripheral blood, but it is understood that relatively few studies in China apply FCM for peripheral blood leukocyte classification in AL. By comparing FCM‐based PBL classification with the current commonly used clinical methods, we hope to further clarify its application value and provide a simpler and more effective method for AL screening and posttreatment monitoring.

In this study, 100 cases of PBLs were classified and counted by FCM and hematology analyzer. The results showed a significant positive correlation between the two methods (*p* < 0.01), indicating that FCM had high accuracy in leukocyte classification and counting and could replace the hematology analyzer in clinical application to some extent.

**FIGURE 6:**
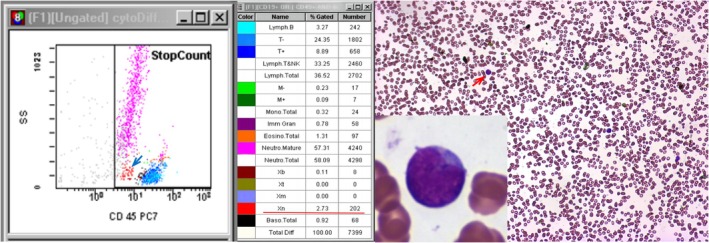
The presence of suspicious blast cell scatter (blue arrow) was detected on the CD45/SS scatter plot during FCM, the flow result suggested the presence of blast cells (red underlined), and microscopic review detected blast cells (red arrow).

There was a significant correlation between FCM and “gold standard” morphological microscopy (manual method) (*p* < 0.01), suggesting that the ability of FCM to detect abnormal white blood cells was comparable to that of the manual method. The manual method detected blast cells in 78 cases (positive rate: 84.7%), while FCM detected them in 80 cases (positive rate: 86.9%). The sensitivity, specificity, and accuracy of FCM were 100%, 85.7%, and 97.8%, respectively. Notably, during this study, 10 specimens were initially flagged by FCM for having suspicious blast cell scatterers on the cd45/ss scatter plot (Figure 6), even though no blast cells were detected by morphological microscopy. Owing to the excellent performance of FCM, it detects blast cells missed by microscopy, and potential medical disputes have been averted. After careful review by senior morphological inspectors, the existence of blast cells was confirmed, though their proportions were very low. The missed detection might be attributed to the reduction of PBLs or changes in leukocyte morphology caused by AL treatment, which made cell identification challenging [19]. It could also be due to the lack of experience among microscopic examination staff. To improve the detection of such low‐proportion cells using morphology, extended and more intensive training or review by more experienced inspectors would be required. However, this would increase the workload and prolong the turnaround time. While FCM can detect 20,000 cells or more at a time, which helps detect blast cells in low white blood cell count samples. Additionally, FCM uses cellular immune markers [20], which can increase the specificity of white blood cell recognition without reducing the sensitivity, allowing it to detect blast cells that may be difficult to identify by morphological microscopy. However, FCM did mistakenly identify blast cells in two cases, and one case was a patient with lymphoma and leukemia. According to empirical analysis, the reason may be that the CD45 expression of the lymphoma cells was weaker than that of normal lymphocytes. This led to a leftward shift of the lymphoid population on the cd45/ss scatter plot, causing the cells to be misclassified as blast cells. The other case may have involved nonspecific antibody binding.

FCM subdivided the blast cells into XB, XT, XM, and XN. Through consistency analysis with the final clinical diagnosis of leukemia type, the kappa value was 0.834, indicating good consistency. This can provide an earlier reference direction for clinical diagnosis and offers valuable hints for developing subsequent bone marrow testing. As a result, unnecessary diagnostic tests may be reduced, thereby lowering the economic burden on patients. In addition, this study compared the correlation between the detection rates of FCM peripheral blood blast cells and bone marrow blast cells in posttreatment patients. While different leukemia patients can present different manifestations of blood and bone marrow fluid due to the presence of the bone marrow‐blood barrier, Spearman analysis showed a significant correlation between the two (*r*s = 0.860, *p* < 0.01). Although bone marrow examination remains irreplaceable in the diagnosis and posttreatment monitoring of AL, we think that in some special cases, such as when bone marrow necrosis caused by bone marrow puncture leads to unrecognizable cells or when bone marrow suppression after chemotherapy results in bone marrow puncture failure, FCM could be used to detect PBLs as a supplementary test. This approach can also be considered when the patients urgently need to understand disease progression due to complications and unstable vital signs after treatment. Using FCM in these situations may reduce the need for frequent bone marrow punctures during posttreatment monitoring.

This study demonstrates that multiparameter FCM provides accurate and rapid classification of peripheral blood leukocytes, with excellent sensitivity for blast detection. It strongly correlates with bone marrow findings, offering a valuable noninvasive tool for acute leukemia screening and posttreatment monitoring, particularly when bone marrow puncture is difficult or inconclusive. Therefore, it is worthy of further promotion in clinical practice.

## Author Contributions

Conceptualization: Hong Lu and Jingjing Qian. Supervision: Jingjing Qian. Data curation: Penghua Yan and Lianfeng Wu. Writing – original draft: Penghua Yan and Jingjing Qian. Review and editing: Xiangyang Lin and Hong Lu. Investigation: Penghua Yan, Hong Lu, and Jingjing Qian. All authors read and approved the final manuscript.

## Funding

This study was funded by the Wenzhou Science and Technology Plan, China (Y2020750). This study was funded by Key Laboratory of Clinical Laboratory Diagnosis and Translational Research of Zhejiang Province (2022E10022).

## Conflicts of Interest

The authors declare no conflicts of interest.

## Data Availability

The datasets used and/or analyzed during the current study are available from the corresponding author upon reasonable request.
